# 

**Published:** 1895-02

**Authors:** 


### CONTENTS—FEBRUARY, 1895.—VOL. X., NO. 8.

ORIGINAL COMMUNICATIONS—
The Treatment of Minor Railway Injuries. A. L. Hathcock, M. D., Palestine . . . . . 385
Castration in Hypertrophy of the Prostate Gland. University Medical Magazine . . . . . 390
Catarrh and its Location in the Human Body. W. W. Pugh, M. D. . 393
CORRESPONDENCE—
Diphtheria and Membranous Croup. L. W. Hollis, M. D.. Abilene . 394
ABSTRACTS AND SELECTIONS—
Reminiscences of Dr. J. Marion Sims in Paris. Edmond Souchon, M. D., New Orleans . . . . . 399
Is it the Beginning of the End? James Weir, M. D., Owensburo, Ky. 405
EDITORIAL—
A Specious Plea . . . . . 417
The Wooten Bill . . . . . 424
MEDICAL NEWS AND MISCELLANY—
Interesting Paragraphs . . . . . 426
BOOK NOTICES— . . . . . 427
PUBLISHERS' NOTES . . . . . 433

